# Receipt of a psychosocial intervention for prenatal anxiety and risk of perinatal intimate partner violence in Pakistani women

**DOI:** 10.1371/journal.pgph.0005700

**Published:** 2025-12-30

**Authors:** Soim Park, Abid Malik, Anne Batchelder, Ahmed Zaidi, Najia Atif, Atif Rahman, Pamela J. Surkan

**Affiliations:** 1 Department of International Health, Johns Hopkins Bloomberg School of Public Health, Baltimore, Maryland, United States of America; 2 Health Services Academy, Islamabad, Pakistan; 3 Human Development Research Foundation, Islamabad, Pakistan; 4 Johns Hopkins University School of Nursing, Baltimore, Maryland, United States of America; 5 Institute of Population Health, University of Liverpool, Liverpool, United Kingdom; The University of Newcastle Australia: University of Newcastle, AUSTRALIA

## Abstract

Mental health and intimate partner violence (IPV) are urgent public health problems in the perinatal period. We examined whether a Cognitive Behavioral Therapy (CBT) intervention designed to lower prenatal anxiety led to differing risk of perinatal IPV between the intervention and control arms. We investigated if social support or spousal relationship quality in the third trimester mediated this association. The analysis included 755 pregnant Pakistani women with at least mild anxiety participating in a randomized clinical trial, which was conducted from April 2019 to October 2022. Intervention arm participants received up to six CBT-based treatment sessions (and up to five booster sessions), while controls received enhanced usual care. After implementing multivariate imputation using chained equations (MICE), we conducted log binomial regression analyses to assess the risk ratios (RRs) for any, physical, and emotional IPV in the intervention versus control arms. We assessed the mediating roles of spousal relationship and social support in the third trimester through causal mediation analyses. 27.7% of participants experienced any perinatal IPV. Compared to women in the control group, those in the intervention arm had significantly lower risk of reporting any IPV (adjusted risk ratio (aRR)=0.73, 95% confidence interval (CI): 0.58-0.91), physical IPV (aRR = 0.63, 95% CI:0.45-0.89), and emotional IPV (aRR = 0.74, 95% CI:0.56-0.93). These associations were partially mediated by spousal relationship quality and social support available in the third trimester. Receipt of a CBT intervention aiming to reduce anxiety was inversely associated with risk of perinatal IPV, likely through improving social relationships during the pregnancy.

## 1. Introduction

Nearly 40% of women in the world have been exposed to intimate partner violence (IPV) [1], with women in South Asia having the highest lifetime prevalence [2]. Globally, about 20% of perinatal women report experiencing IPV in the past year [1], and risk factors include low socioeconomic status, low household income, low education attainment of husband, and the husband having an unskilled manual occupation [3,4]. Notably, the risk of IPV is heightened for women during the perinatal period [5,6]. IPV tends to reoccur during pregnancy and postpartum period and often increases in severity over time [7,8]. During the perinatal period, both women and their intimate partners may face challenges in managing uncertainty and regulating emotions, along with relationship dissatisfaction and lack of social support, which together increase their risk of IPV [9–12]. Experiencing IPV in the perinatal period is associated with poor health outcomes for both mothers and their infants [13,14].

Moreover, IPV and anxiety are closely interconnected. IPV is a well-established risk factor for perinatal anxiety [15]. IPV and anxiety share some common risk factors, including childhood abuse, stress, poor relationship dynamics with one’s spouse, and lack of social support [16–20]. Care strategies for individuals experiencing IPV and anxiety also overlap in several ways, such as the need for strong rapport with providers, person-centered and trauma-informed care, stress management, and social support resources [21,22]. In Pakistan, about 35–49% of pregnant women suffer from anxiety [23,24], which may persist and lead to postpartum depression and anxiety. While prevalence estimates for perinatal IPV are not available in Pakistan, it is estimated that about one-third of ever-married Pakistani women have experienced IPV during their lifetimes [2,25].

Psychological therapies, such as Cognitive Behavioral Therapy (CBT), are effective and acceptable interventions for perinatal anxiety [22]. Specifically, the delivery of psychological therapies by non-specialist providers (NSPs) is an effective strategy to reach those who need services and to bridge the treatment gap in low- and middle-income countries (LMICs) [26]. While in other settings medication is recommended to treat moderate-severe perinatal depression and anxiety [27], pharmacological treatments are not feasible in Pakistan where the ratio of psychiatrists to the population is 0.19 per 100,000 residents [28].

Due to the epidemic proportions of IPV in South Asia, its importance in the perinatal period, and the stigma attached to it, it is imperative to identify non-stigmatized strategies to address the problem. Our main intervention target was to reduce symptoms of anxiety among pregnant women, which led to both lower postpartum depression and symptoms of anxiety [29]. While randomized trials have focused on reducing IPV using NSPs in the South Asian region [30,31], none to our knowledge have had reducing anxiety as a major aim of their intervention. Considering the interconnectivity of mental health conditions and IPV, this type of intervention could also affect the risk of IPV. As an exploratory study, our primary aim was to investigate whether a CBT-based intervention for symptoms of anxiety during pregnancy would show differences in the risk of IPV during the perinatal period. Secondarily, we examined if spousal relationship quality and social support from family and friends in the third trimester mediated this association.

## 2. Materials and methods

### 2.1. Ethics statement

Written informed consent was obtained prior to screening and data collection. This study was approved by the Institutional Review Boards (IRBs) of Rawalpindi Medical University (IRB/RMU-20/12/20190), the Human Development Research Foundation (IRB/001/2017), the Johns Hopkins Bloomberg School of Public Health (IRB No. 00009177), and a US National Institute of Mental Health appointed Data Safety Monitoring Board.

### 2.2. Trial design

This was a phase 3, two-arm, single-blind, randomized controlled trial (RCT) with a 1:1 allocation ratio.

### 2.3. Participants, eligibility criteria, and setting

**Fig 1:**
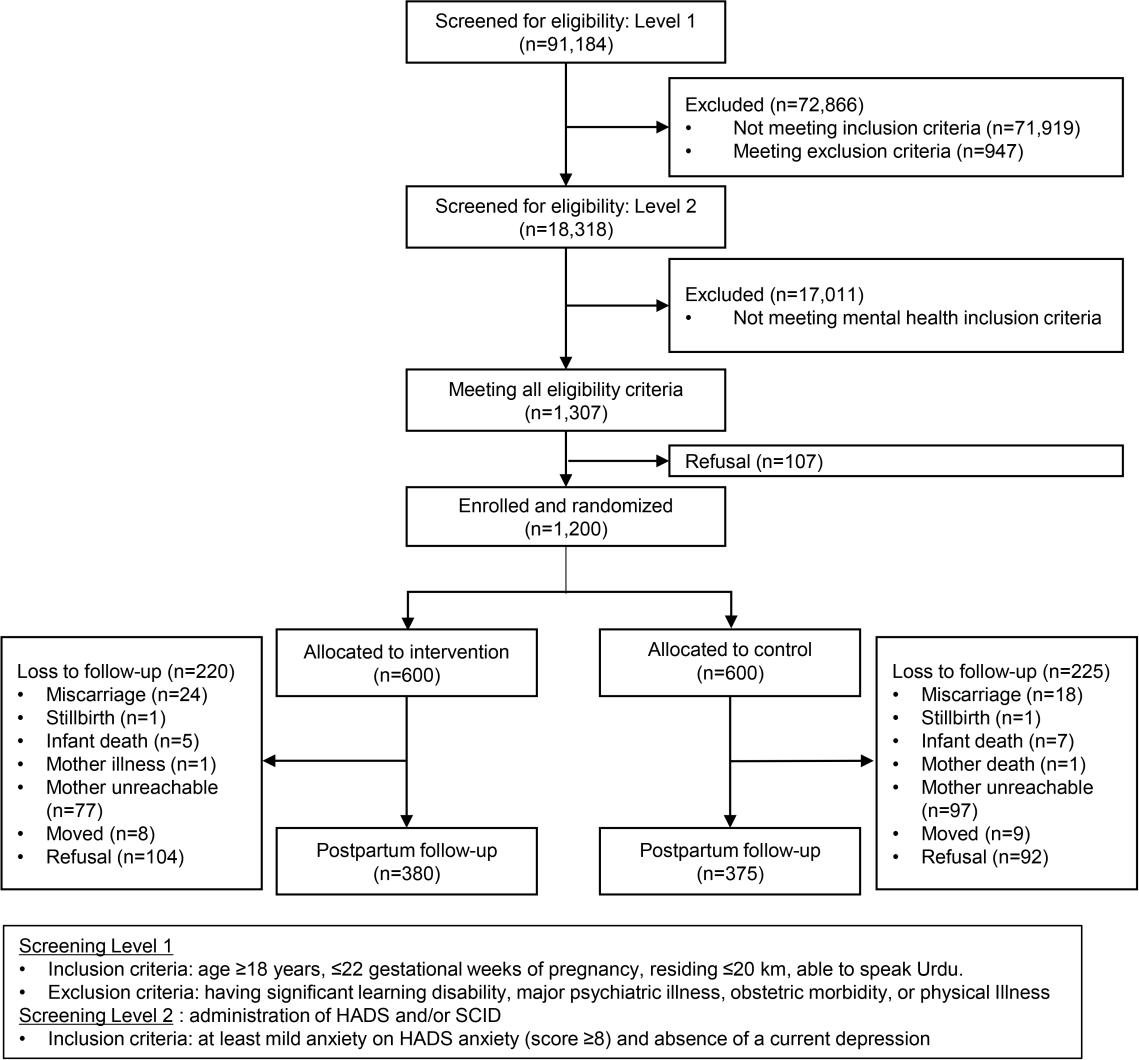
Participant flowchart.

Between April 2019 and January 2022, we enrolled 1,200 pregnant Pakistani women in an RCT offering a CBT-based intervention to reduce prenatal anxiety (clinicaltrial.gov: NCT03880032) [32]. All women finished the final six-week postpartum visit by October 2022. Pregnant women were recruited when seeking obstetric care at a large public tertiary hospital in Rawalpindi District. Eligibility criteria were gestational age ≤ 22 weeks, age ≥ 18, living within 20 kilometers of the study hospital, and being able to speak Urdu. Women who consented to participate were administered an Urdu-adapted and validated Hospital Anxiety and Depression Scale (HADS) [33] and an Urdu-translated Structured Clinical Interview for DSM-5. We included women with at least mild anxiety (≥8 on the HADS anxiety subscale) without a diagnosis of clinical depression. Exclusion criteria were self-reported current or past mental disorders or psychiatric care (for details, see Surkan et al., (2020)). Participants completed questionnaires at baseline, in the third trimester, and at six-weeks postpartum. In this analysis, we included 755 women who completed the postpartum survey (**Fig 1**). The primary outcomes of the parent study included common mental health disorders (CMDs; i.e., depression and anxiety), and secondary outcomes were birth outcomes. These findings are published elsewhere [29,34]. The current study, focusing on whether the HMHB intervention is associated with the risk of perinatal IPV, was an *ad hoc* analysis of the RCT, along with other *ad hoc* studies that examined how the intervention influenced breastfeeding, functional disability, maternal-infant bonding, and child development [35–38].

### 2.4. The Intervention

Happy Mother - Healthy Baby (HMHB) is a prenatal anxiety intervention adapted from the evidence-based Thinking Healthy Program (see Atif et al., (2020)). The intervention consisted of six core sessions of one-on-one CBT delivered by NSPs who had completed a two-year bachelor’s degree and a two-year master’s degree in psychology (equivalent to a bachelor’s degree outside of Pakistan) but without prior clinical experience. The first five weekly sessions started as soon as the woman enrolled in the study. The final core session was offered in the third trimester. The intervention used key CBT elements including thought challenging, behavior activation, relaxation exercises, and problem solving as well as engagement of the family to promote support. The core sessions were 60 minutes. A maximum of six thirty-minute booster sessions were offered to all women in intervention arm between the fifth and sixth core sessions, which were timed to coincide with their routine antenatal visits.

The first session aimed to build rapport between the NSPs and the participant, to introduce the three steps of thinking and acting healthy. The second session focused on woman’s overall well-being (e.g., diet, resting) and relaxation exercises to manage stress. The third session concentrated on the woman’s relationships to improve social support. The fourth session aimed to promote woman’s attachment with her unborn baby. The NSP and participant also discussed anxieties around labor and delivery. The fifth session involved reviewing the previous sessions and maintaining the helpful practices already learned. The final core session included discussion about potential problems in the postpartum period. Family members (e.g., spouse, mother-in-law) were specifically invited to the first, third, and sixth sessions to negotiate support during and after pregnancy (the main topic of the third session being on getting social support), but they were welcomed to attend any sessions. Social support was incorporated into all core sessions; thus, family members who attended any sessions may have learned about the importance of social support during and after pregnancy. The booster sessions reinforced health messages and encouraged problem management strategies to handle stress [39]. While women’s well-being and empowerment remained consistent themes throughout all sessions, the third session raised culturally sensitive information regarding different types of IPV (emphasizing its harmful effects on women and babies during the perinatal period). Anxiety was monitored in each session by reviewing a visual scale of a participant’s emotional state. If the participant was consistently very anxious or if her self-reported mood worsened, she was referred to specialist care.

During a national COVID-19 lockdown in Pakistan that coincided with data collection, all in-person study activities were completely suspended. As a result, some women recruited just before the lockdown had to participate in most or all HMHB sessions by phone, although the original study design was to provide only face-to-face sessions. In our qualitative study exploring the delivery methods for the intervention sessions, we found in-person sessions enhanced the rapport that participants had with the therapists, such that possibly for some women phone delivery contributed to dropout [40]. However, some participants preferred a combination of in-person and phone sessions due to the ease of scheduling, the convenience of not having to leave home, or not needing to be accompanied when visiting the hospital [40].

### 2.5. Measures

Data were self-reported using the Open Data Kit. Exposure to perinatal IPV was measured only at six-weeks postpartum, whereas spousal relationship quality and social support were measured at baseline, in the third trimester, and at six-weeks postpartum.

- Perinatal intimate partner violence

At six-weeks postpartum, we measured exposure to IPV occurring since the beginning of the pregnancy, referred to as perinatal IPV. A 10-item scale from the Pakistan Demographic and Health Survey (DHS) was used to assess exposure to physical (7 items) and emotional (3 items) IPV [25]. Each item asks whether the woman was exposed to specific IPV events (e.g., pushed, slapped, insulted), with answer options of ‘yes’ and ‘no.’ Women who answered ‘yes’ to any item were classified as exposed to IPV.

- Relationship quality with the spouse

To assess relationship quality with a spouse, we adapted six items from the MacArthur battery [41]. It was originally created to evaluate whether older adults’ social network ties provide emotional or instrumental support, or if they are the source of conflict and/or demand [41]. Adapting these items to the spouse, we asked about the frequency of woman’s spouse in providing emotional support, instrumental support, or the spouse becoming source of conflict/demand (each subscale having two items). Answer categories were on a five-point Likert scale ranging from 0 (never) to 4 (frequently). When calculating the mean of the full scale, we reverse scored the conflict/demand domain, so that high scores indicated good spousal relationship quality. We used the relationship quality measure that was assessed in the third trimester as a potential mediator.

- Social support from family or friends

We used a version of the 12-item Multidimensional Scale of Perceived Social Support that was validated in Pakistan to measure social support [42]. A series of questions asks about participants’ level of agreement to the perceived availability of social support from a significant other, family, and friends (each subscale using four items). Answer options were on a five-point Likert scale ranging from 0 (very strongly disagree) to 4 (strongly agree). Higher scores indicated greater perceived social support. To avoid potential overlap with spousal relationship quality, we excluded support from the significant other, and used the mean of the two subscales (support from family and friends) as a full scale. Social support assessed in the third trimester was included as a potential mediator.

- Covariates

For baseline participant characteristics, we included women’s age, education level (≤ or > middle school), HADS anxiety and depression scores, gravidity (primigravida vs. multigravida), previous pregnancy loss, exposure to any physical violence in the past three months, relationship quality scores, and social support scores. For household characteristics, we included husband’s education level (≤ or > middle school), husband’s employment, family structure (nuclear, joint, or extended), and monthly household income, which was categorized into low (<20,000 Pakistani Rupee (PKR)≈US $124, as of January 1, 2021), medium (20,000–35,000 PKR), and high (>35,000 PKR ≈ US $218).

### 2.6. Sample size calculation

The original sample size in the parent study was calculated assuming 30% of prevalence of CMDs in the study population. To achieve 85% power to detect a 30% reduction in CMDs while expecting 30% attrition after enrolment, our target was to enroll total of 1,200 women [29]. We conducted a *post hoc* power analysis using Fisher’s exact test based on the prevalence of IPV in our sample and the sample size available for this analysis. The power in the *post hoc* analysis was estimated to be 57.5%, with the Type I error rate set at 0.05.

### 2.7. Randomization and blinding

Before the baseline assessment, all enrolled participants were randomized equally into the intervention or control group. The trial statistician in the United States used a pseudo random-number generator to assign women to the two arms, based on randomly permuted blocks of size 4, 8, 12, and 16. The list of arm assignment was printed in order, with the steps of the sequence stored separately in opaque envelopes. When an eligible woman provided consent to participate, the study staff pulled and opened the next available envelope and recorded the arm assignment. This procedure continued until we enrolled 600 women in each arm. Only the statistician in the United States and the data manager in Pakistan (located outside of the study hospital) could access the randomization codes. All outcome assessors were blind to the arm allocation of the participants.

### 2.7. Statistical analysis

Following the intent-to-treat principle to compare women assigned to the intervention and control groups, we calculated descriptive statistics stratified by treatment arm. Because 17.6% of the sample had missingness on income, social support and/or relationship quality assessed in the third trimester, we conducted multivariate imputation using chained equations (MICE) by creating 50 imputations [43]. For reproducibility, we generated a random number as a seed [44]. To calculate risk ratios (RRs), we used log binomial regression or Poisson regression with robust variance (when log binomial models failed to converge) [45]. We first conducted bivariate analyses between baseline characteristics and IPV. Variables associated with IPV at p < 0.05 were included as potential confounders in the multiple regression analyses using log binomial regression or Poisson regression with robust variance. Adjusting for exposure to any physical violence, monthly household income, relationship quality and social support at baseline, we further assessed whether receiving the intervention was associated with perinatal IPV. For the per-protocol analysis, we categorized women into those who received no intervention sessions (n = 424 including 375 women in the control arm), a low-dose group (those who received 1–5 core sessions; n = 111), and a high-dose group (women who received either 1) five core sessions and at least one booster session; 2) six core sessions; or 3) six core sessions and at least one booster session; n = 220)), and investigated the dose-response relationship between sessions received and IPV. These analyses were conducted using STATA 15.1 (StataCorp, College Station, TX). All statistical tests were two-sided at the p < 0.05 level.

Lastly, we ran causal mediation analyses [46] separately for each of two variables related to social relationships (spousal relationship quality and social support assessed in the third trimester), controlling for exposure to physical violence, income, relationship quality and social support at baseline. If the two mediators captured independent pathways from treatment to outcome, these analyses could be interpreted causally. Causal mediation analysis with multiply imputed data was performed using the “intmed” package in R (R Foundation for Statistical Computing, Vienna, Austria).

## 3. Results

Women’s median age was 25 years (Table 1). About 57.1% of the sample had more than a middle school education, and the majority (92.7%) lived with husband. Median HADS anxiety and depression scores were 11 and 7, respectively. Only 27.8% were primigravida and 43.4% had experienced a prior pregnancy loss. At baseline, 10.9% reported experiencing physical violence (not specifically perpetrated by partner) in the past three months. Also at baseline, women in intervention arm were more likely to report experiencing physical violence in the past three months (13.4% vs. 8.3% control). Baseline spousal relationship and social support median scores were 2.8 and 2.1, respectively. When spousal relationship and social support were assessed in the third trimester, women in intervention arm showed better spousal relationships and more social support from family and friends. About 59.1% of husbands had more than a middle school education, and most husbands were employed (95.2%). One-third of women were living within a nuclear family, and 89.9% had monthly household incomes of ≤35,000 PKR.

**Table 1:** Descriptive statistics (n = 755).

	Total (n = 755)	Intervention group (n = 380)	Control group (n = 375)
	**n (%)**	**n (%)**	**n (%)**
**Participant characteristics**			
Age, median (Q1-Q3)	25.0 (22.0-28.0)	25.0 (22.0-28.0)	25.0 (22.0-28.0)
Education level			
≤ Middle school (≤8 years)	324 (42.9)	170 (44.8)	154 (41.1)
> Middle school (>8 years)	431 (57.1)	210 (55.3)	221 (58.9)
Living with husband (vs. not living with husband)	700 (92.7)	348 (91.6)	352 (93.9)
HADS anxiety score, median (Q1-Q3)	11.0 (10.0-12.0)	11.0 (10.0-12.0)	11.0 (10.0-12.0)
HADS depression score, median (Q1-Q3)	7.0 (5.0-9.0)	7.0 (5.0-9.0)	7.0 (5.0-8.0)
Gravidity			
Multigravida	545 (72.2)	281 (73.6)	264 (70.4)
Primigravida	210 (27.8)	99 (26.1)	111 (29.6)
Previous pregnancy loss (vs. none)	328 (43.4)	172 (45.3)	156 (41.6)
Exposure to any physical violence (vs. none)	82 (10.9)	51 (13.4)	31 (8.3)
Baseline relationship quality score, median (Q1-Q3)	2.8 (2.3, 3.3)	2.8 (2.3, 3.3)	2.8 (2.2, 3.3)
Baseline social support score, median (Q1-Q3)^†^	2.1 (1.5, 3.0)	2.1 (1.5, 3.0)	2.1 (1.4, 3.0)
Third trimester relationship quality score, median (Q1-Q3)^‡^	3.0 (2.3, 3.3)	2.8 (2.2, 3.3)	3.0 (2.7, 3.5)
Third trimester social support score, median (Q1-Q3)^†‡^	2.4 (1.6, 3.0)	2.0 (1.4, 3.0)	2.8 (1.9, 3.0)
**Household characteristics**			
Husband’s education level			
≤ Middle school (≤8 years)	309 (40.9)	161 (42.4)	148 (39.5)
> Middle school (>8 years)	446 (59.1)	219 (57.6)	227 (60.5)
Husband employed (vs. unemployed)	719 (95.2)	364 (95.8)	355 (94.7)
Family structure^^^			
Nuclear	245 (32.5)	126 (33.2)	119 (31.7)
Joint	254 (33.6)	124 (32.6)	130 (34.7)
Extended	256 (33.9)	130 (34.2)	126 (33.6)
Monthly household income (Pakistan Rupees (PKR))			
Low (<20,000)	356 (47.2)	175 (47.6)	181 (49.2)
Middle (20,000–35,000)	322 (42.7)	160 (43.5)	162 (44.0)
High (>35,000)	58 (7.7)	33 (9.0)	25 (6.8)
Unknown	19 (2.5)	12 (3.2)	7 (1.9)

*Note*. Column percentages are presented.

^†^To prevent overlap with relationship quality with spouse, we excluded a subscale measuring support from significant other. This score is an average of two subscales: support from family and support from friends.

^‡^Includes 637 participants who completed the assessment in the third trimester.

^Joint household includes family living with in-law parents, and extended housdhold includes family living with in-law parents, siblings, and their family members.

In bivariate analyses, exposure to physical violence in pregnancy, worse spousal relationship quality, and less social support at baseline were associated with an increased risk of perinatal IPV (p-values <0.001; S1 Table). Having a medium or high income was inversely associated with IPV exposure (p-values = 0.04).

Women in the control arm were significantly more likely to experience IPV in the perinatal period (31.2% vs. 24.2% any IPV; 16.5% vs. 11.8% physical IPV; and 28.5% vs. 22.4% emotional IPV; Table 2). Adjusting for exposure to physical violence, income, relationship quality, and social support at baseline, women in the intervention arm had a significantly lower risk of reporting any IPV (adjusted risk ratio (aRR)=0.73, 95% confidence interval (CI): 0.58-0.91), physical IPV (aRR = 0.63, 95% CI: 0.45-0.89), and emotional IPV (aRR = 0.74, 95% CI: 0.56-0.93) compared to those in the control arm.

**Table 2:** Associations between treatment and perinatal IPV (n = 755).

	Total (n = 755)	Intervention group (n = 380)	Control group (n = 375)	Unadjusted	Adjusted^a^
Outcome		n (%)	n (%)	RR (95% CI)	RR (95% CI)
Any IPV	209 (27.7)	92 (24.2)	117 (31.2)	0.78 (0.61, 0.98)*	0.73 (0.58, 0.91)**
Physical IPV	107 (14.2)	45 (11.8)	62 (16.5)	0.72 (0.50, 1.02)	0.63 (0.45, 0.89)**
Emotional IPV	192 (25.4)	85 (22.4)	107 (28.5)	0.78 (0.61, 1.00)	0.74 (0.56, 0.93)*

^a^Adjusted for baseline characteristics, including exposure to physical violence, monthly household income, relationship quality with spouse, and social support.

*Note*. Risk ratios were calculated between intervention group (vs. control) and outcomes of interests.

*p < 0.05, **p < 0.01, ***p < 0.001.

**Fig 2:**
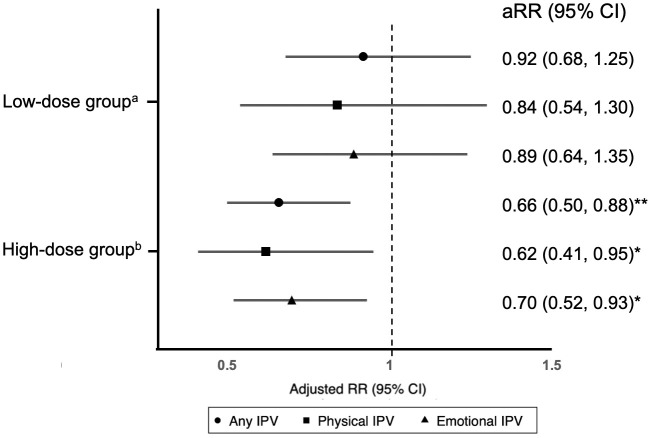
Adjusted risk ratios (RRs) for exposure to perinatal intimate partner violence (IPV) by the number of intervention sessions received, compared to women who received no intervention sessions. Note: The reference is having received no intervention. RRs are adjusted for baseline characteristics, including exposure to physical violence, monthly household income, relationship quality with spouse, and social support. a The low-dose group includes women who received 1–5 intervention sessions. b The high-dose group includes women who received more than five sessions—women who received five core sessions and at least one booster session, six core sessions without any booster sessions, or six core sessions and at least one booster session. *p < 0.05, **p < 0.01, ***p < 0.001.

Among the 331 women who attended at least one session, 81 participated in booster sessions between the 5^th^ and 6^th^ sessions in third trimester. On average, women who participated in booster sessions had on average 1.72 booster sessions (SD = 0.99) in addition to at least five core sessions. In the per-protocol analysis, women in low-dose or high-dose groups tended to be exposed to less IPV, but the differences were not significant (Table 3). After adjustment, women who received more than 5 sessions (either core or booster sessions) had lower risk of reporting IPV, compared to women who received no intervention sessions (aRR = 0.66, 95% CI: 0.50-0.88 any IPV; aRR = 0.62, 95% CI: 0.41-0.95 physical IPV; and aRR = 0.70, 95% CI: 0.52-0.93 emotional IPV; Fig 2). No significant association between the intervention and risk of perinatal IPV was found for women who received 1–5 sessions without booster sessions.

**Table 3:** Estimated intervention effects by the number of intervention sessions received (n = 755).

	Intensity of intervention sessions received			
	No intervention (n = 424)^a^	Low-dose^b^ (n = 111)	High-dose^c^ (n = 220)	
Outcomes	n (%)	n (%)	n (%)	p-value^d^
Any IPV	128 (30.2)	34 (30.6)	47 (21.4)	0.05
Physical IPV	66 (15.6)	17 (15.3)	24 (10.9)	0.26
Emotional IPV	117 (27.6)	30 (27.0)	45 (20.5)	0.13

^a^Includes women in control arm (n = 346) and those in intervention arm who did not receive any therapy sessions (n = 44).

^b^The low-dose group includes women who received 1–5 intervention sessions.

^c^The high-dose group includes women who received more than five sessions—women who received five core sessions and at least one booster session, six core sessions without any booster sessions, or six core sessions and at least one booster session.

^d^Chi-squared tests were used to compare the differences in IPV exposure stratified by intervention sessions received.

In the causal mediation analysis, the average causal mediation effect (ACME) through spousal relationship quality in the third trimester was significant for the associations between treatment and three perinatal IPV outcomes (**Table 4**, p-values<0.001). Spousal relationship quality in the third trimester mediated 25%, 23%, and 30% of the total effect of the association between treatment and any IPV, physical IPV, and emotional IPV during perinatal period, respectively. However, social support from family or friends in the third trimester was only a significant mediator between the treatment and emotional IPV (p = 0.03), accounting for 19% of the total effect.

**Table 4:** Estimates of average direct effects (ADE), average causal mediation effects (ACME), and total effects (mediated through social support and relationship quality with spouse in the third trimester) of the association between treatment (i.e., intervention vs. control groups) and exposure to perinatal intimate partner violence (IPV).

Mediators	Effect type	Estimate (95% CI)	p-value	Proportion of total effect mediated
**Any type of IPV**				
Relationship quality in the third trimester	ADE	-0.06 (-0.14, 0.02)	0.139	0.25
	ACME	-0.02 (-0.04, -0.01)	<0.001	
	Total effect	-0.08 (-0.16, -0.01)	0.033	
Social support in the third trimester	ADE	-0.07 (-0.16, 0.01)	0.075	0.14
	ACME	-0.01 (-0.03, 0.00)	0.083	
	Total effect	-0.09 (-0.17, -0.01)	0.028	
**Physical IPV**				
Relationship quality in the third trimester	ADE	-0.05 (-0.11, 0.01)	0.134	0.23
	ACME	-0.01 (-0.03, -0.01)	<0.001	
	Total effect	-0.06 (-0.12, 0.00)	0.037	
Social support in the third trimester	ADE	-0.06 (-0.12, 0.00)	0.054	0.09
	ACME	-0.01 (-0.02, 0.00)	0.208	
	Total effect	-0.06 (-0.12, -0.01)	0.026	
**Emotional IPV**				
Relationship quality in the third trimester	ADE	-0.05 (-0.13, 0.03)	0.220	0.30
	ACME	-0.02 (-0.04, -0.01)	<0.001	
	Total effect	-0.07 (-0.15, 0.00)	0.059	
Social support in the third trimester	ADE	-0.06 (-0.14, 0.02)	0.136	0.19
	ACME	-0.02 (-0.03, 0.00)	0.033	
	Total	-0.08 (-0.15, 0.00)	0.045	

*Note*. All models were adjusted for exposure to physical violence during pregnancy, monthly household income, relationship quality with spouse and social support measured at baseline.

## 4. Discussion

Results suggest that among Pakistani pregnant women with at least mild anxiety, those randomized to the intervention arm were less likely to experience perinatal IPV compared to those in the control arm. This effect was particularly pronounced among women who received a high dose of CBT-based sessions (i.e., more than 5 sessions). Spousal relationship quality in the third trimester partially mediated the associations between the intervention and different types of IPV (any, physical, and emotional IPV), whereas social support from family and friends was a significant partial mediator only for the association between the intervention and emotional IPV.

The existing literature has tended to focus on unidirectional relationship of IPV’s effects on mental health outcomes [47,48] and how reducing IPV can enhance psychological well-being [49]. Although our study does not investigate the causality of whether IPV contributed to pregnant women’s anxiety or not, we found that women who received the intervention to reduce prenatal anxiety were less likely to report perinatal IPV. This is an important finding given that the intervention sessions did not directly target IPV. Instead, HMHB aimed to alleviate anxiety in women by improving pregnant women’s social relationships and sought to provide general information on the harmfulness of IPV. In combination, these efforts resulted in less IPV. Thus, this intervention offers a potentially important strategy that can both reduce IPV and lower anxiety during the perinatal period, while avoiding the stigma of being an IPV intervention [29].

**Fig 3:**
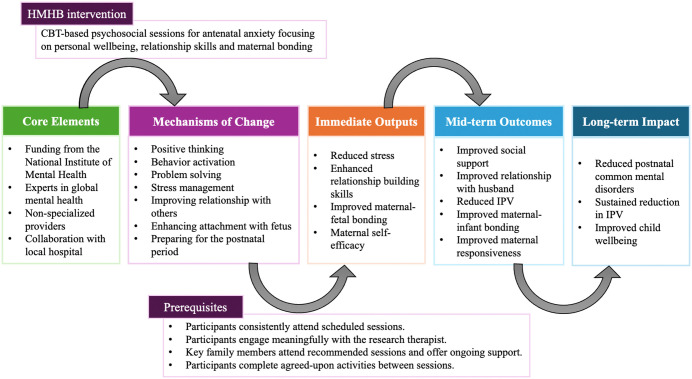
Theory of Change Model for the Happy Mother—Healthy Baby (HMHB) intervention. Abbreviations: HMHB Happy Mother—Healthy baby; IPV Intimate partner violence.

As shown in the impact model that indicates possible relationships between the HMHB intervention, IPV, and CMDs (**Fig 3**), our findings suggest that improving positive spousal relationships can help mitigate perinatal IPV exposure in the context of our CBT-focused intervention. Notably, family members of HMHB participants were encouraged to attend three intervention sessions to help reduce women’s anxiety. Primary care settings are often effective venues for delivering IPV interventions through allied healthcare practitioners, including nurses, social workers, and paramedics [50,51]. While most of these interventions have been conducted in high-income countries [50], a few studies in China and Mongolia (middle-income countries) have aimed to reduce IPV by delivering interventions through midwives, social workers, or other NSPs [30,52,53], which may offer a cost-effective approach in resource-limited settings. Along with various strategies to reduce IPV that have shown to be effective (e.g., targeting perpetrators, supporting victims) [54,55], the WHO highlights the need to strengthen relationship skills among couples through improving interpersonal communication and conflict management [56]. Improving marital relationships is essential, as one meta-analysis identified a negative relationship between marital satisfaction and IPV [57]. While attitude change in perpetrators is important for reducing IPV [55], changes in women’s attitudes and communication skills with their spouses may also help. Another US study suggested that multi-couple intervention sessions reduced IPV recidivism in perpetrators more significantly than individual couple intervention sessions [58]. Future research could evaluate whether developing one of the HMHB sessions into a multi-couple therapy session targeting the expansion of women’s social networks to improve social support could further reduce IPV.

Our finding that social relationships was a strong mechanism through which HMHB appears to be associated with lower perinatal IPV is supported by literature showing that social support from family and friends is a crucial protective factor for women exposed to IPV across contexts [59,60]. Abused women with available social support cope better with the sequelae of IPV, including depression and anxiety [59,60]. Social support from friends has been reported as helpful, alleviating emotional distress and providing tangible support such as childcare [61,62], whereas social support from family has shown inconsistent findings [62–64]. While parents of victimized women can be sources of support that help decrease IPV [64], some women from cultures that accept or tolerate gender-based violence report that close family sometimes advise them to conform to dominant gender roles, not necessarily protecting them against IPV [62,63]. Furthermore, the ability of the woman’s natal family to provide support may be reduced when women live with in-laws or far from their birth families, and in some cases the husband and in-law family control women’s ability to travel to see her natal family [65]. Other research has found that in-laws often support the IPV perpetrator [66], and conflict with in-laws could lead to IPV [67]. About two-thirds of our study participants lived in households with in-law parents or in-law siblings. In this setting, the role of in-law family members is likely to be important to the risk of IPV. Although one of our qualitative studies in this population found that pregnant women living with in-laws tended to feel especially unsupported [65], our mediation analysis suggested that social support from family (other than the husband) and friends still significantly mediated the relationship between getting the intervention and IPV. Nonetheless, we lacked data about which family members/friends provided support. However, given that in-laws are important sources of social support for mothers in Pakistan [65], culturally appropriate strategies to engage them to further reduce the risk of IPV should be developed.

One limitation is that ~37% of participants were lost to follow-up, in part due to the COVID-19 pandemic in which pregnant women feared infection and missed antenatal appointments [68]. We may have lost more women at risk of IPV, including those lacking autonomy to access healthcare without permission. Furthermore, our study may be underpowered for detecting the risk of IPV. Given that we still found the intervention to lower likelihood of IPV in the per protocol analysis, it is possible that a future fully powered study may be also able to detect an effect in the intent-to-treat analysis. Because we lacked information on IPV at baseline, we were only able to adjust for exposure to violence generally (as a proxy for IPV) at this data collection point, rather than for IPV specifically. Furthermore, IPV was assessed only during the postpartum period by asking about any exposure throughout the perinatal period. As a result, we were unable to determine the exact timing of the exposure. Although the majority of women are likely to have been exposed to recurrent IPV [7,8], there is a chance that some women may have been exposed to IPV only before receiving the intervention. This limitation regarding temporality, however, would be expected to bias the results towards the null. Nonetheless, given these limitations, these findings should be considered exploratory and need confirmation. Future studies that evaluate the risk of IPV separately at baseline (during pregnancy) and postpartum would provide more definitive evidence regarding the effect of the intervention on IPV risk. Our participants represented a catchment population from a public hospital in an urban Pakistan, limiting the study’s generalizability to similar populations. Finally, in the causal mediation analysis, we treated the two mediators (spousal relationship and social support) independently, however it is likely that the mediators are themselves causally interconnected. Although this interconnectivity is not represented in our mediation models, we interpret the results as suggestive of the hypothesis that the two mediators jointly mediate the total effect. A key strength is that we used IPV measures from the Pakistan DHS and sought to fill the gap in the literature on IPV in LMICs.

Overall, an intervention designed to reduce prenatal anxiety that included a component to improve the pregnant women’s social relationships, showed a lower prevalence of IPV among women in intervention arm, compared to those in control arm. This association was mediated by the spousal relationship and social support from family and friends. Notably, women who received a high-dose HMHB reported lower risk of IPV, compared to those receiving no intervention or a low-dose of the intervention. These findings are hypothesis generating in that they suggest that CBT-based interventions not specifically designed to mitigate the risk of IPV may have beneficial effects, even without explicitly incorporating the components aimed at reducing IPV. The findings are only transferrable to similar low- or middle-income pregnant women who have prenatal anxiety, living in low-income settings like Pakistan. To confirm the effectiveness of the intervention on IPV, future research should investigate IPV measured at multiple time points, test whether NSPs with lower educational attainment can effectively deliver the program, and study this relationship among larger samples in diverse geographical areas in other LMICs where IPV is prevalent.

## Supporting information

S1 Table Bivariate regression analyses to examine factors related to exposure to intimate partner violence (IPV) at postpartum (n = 755). (DOCX)

S1 File CONSORT checklist. (DOCX)

S2 File Inclusivity in global research. (DOCX)
