# Supplementary material for: Receipt of a psychosocial intervention for prenatal anxiety and risk of perinatal intimate partner violence in Pakistani women
Source: PLOS Glob Public Health. 2025 Dec 30;5(12):e0005700. doi: 10.1371/journal.pgph.0005700 (PMC12753053; doi:10.1371/journal.pgph.0005700)
Supplement: S1 Table — (DOCX) [file pgph.0005700.s001.docx]

**Supplementary Table 1**. Bivariate regression analyses to examine factors related to exposure to intimate partner violence (IPV) at postpartum (n=755)

|  | Any type of IPV | p-value |
| --- | --- | --- |
|  | RR (95% CI) |  |
| **Participant characteristics** |  |  |
| Age, mean (SD) | 1.00 (0.98, 1.03) | 0.81 |
| Education level |  |  |
| ≤ Middle school (≤8 years) | Ref |  |
| > Middle school (>8 years) | 0.84 (0.66, 1.05) | 0.13 |
| Living with husband (vs. not living with husband) | 1.09 (0.69, 1.75) | 0.71 |
| HADS anxiety score, mean (SD) | 1.01 (0.96, 1.08) | 0.65 |
| HADS depression score, mean (SD) | 1.02 (0.98, 1.06) | 0.45 |
| Gravidity: Primigravida (vs. multigravida) | 0.93 (0.71, 1.21) | 0.57 |
| Previous pregnancy loss (vs. none) | 1.04 (0.83, 1.32) | 0.72 |
| Exposure to any physical violence (vs. none) | 2.38 (1.89, 3.00) | <0.001 |
| Relationship quality score at baseline, mean (SD) | 0.69 (0.63, 0.77) | <0.001 |
| Social support score at baseline, mean (SD)^†^ | 0.72 (0.65, 0.80) | <0.001 |
| **Household characteristics** |  |  |
| Husband’s education level |  |  |
| ≤ Middle school (≤8 years) | Ref |  |
| > Middle school (>8 years) | 0.88 (0.70, 1.11) | 0.28 |
| Husband employed (vs. unemployed) | 0.75 (0.48, 1.18) | 0.02 |
| Family structure^‡^ |  |  |
| Nuclear | Ref |  |
| Joint | 0.85 (0.65, 1.12) | 0.25 |
| Extended | 0.80 (0.60, 1.05) | 0.11 |
| Monthly household income (PKR), mean (SD) |  |  |
| Low (<20,000) | Ref |  |
| Medium (20,000-35,000) | 0.77 (0.61, 0.98) | 0.04 |
| High (>35,000) | 0.55 (0.31, 0.98) | 0.04 |

Note: Multiple imputation using chained equations (MICE) was conducted to take account for missing values, and risk ratio (RRs) were calculated using log binomial regression or Poisson regression with robust variance.

^†^ To prevent overlap with relationship quality with spouse, we excluded a subscale measuring support from significant other. This score is an average of two subscales: support from family and support from friends.

^‡^ Non-nuclear household includes family living with in-law parents, or family living with in-law parents, siblings, and their family members.
